# Supplementary material for: Effects of Copper on Legionella pneumophila Revealed via Viability Assays and Proteomics
Source: Pathogens. 2024 Jul 3;13(7):563. doi: 10.3390/pathogens13070563 (PMC11279431; doi:10.3390/pathogens13070563)
Supplement: Supplementary file 1 [file pathogens-13-00563-s001.zip › pathogens-3018301-supplementary.pdf]

Supplemental Information

Effects of Copper on Legionella pneumophila Revealed via Viability Assays and Proteomics

Yang Song 1,2,*, Didier Mena-Aguilar 3,4, Connor L. Brown 5, William J. Rhoads 1,6, Richard F. Helm 7, Amy Pruden 1,* and Marc A. Edwards 1

1 Civil and Environmental Engineering, Virginia Tech, 1145 Perry St., 418 Durham Hall, Blacksburg, VA 24061, USA

2 Utilities Department, 316 N. Academy St., Town of Cary, Cary, NC 27513, USA

3 Biochemistry, Virginia Tech, 340 W Campus Dr, Blacksburg, VA 24060, USA

4 Department of Biochemistry, University of Nebraska-Lincoln, N106, The Beadle Center, Lincoln, NE 68588, USA

5 Genetics, Bioinformatics, and Computational Biology, Virginia Tech, Steger Hall, Blacksburg, VA 24061, USA

6 Black & Veatch, 8400 Ward Pkwy, Kansas City, MO 64114, USA

7 Department of Biochemistry, Virginia Tech, 1015 Life Science Circle, 211B Steger Hall, Blacksburg, VA 24061, USA

* Correspondence: ys117@vt.edu (Y.S.); apruden@vt.edu (A.P.)

Number of Pages: 17

Number of Figures: 4

Number of Tables: 5

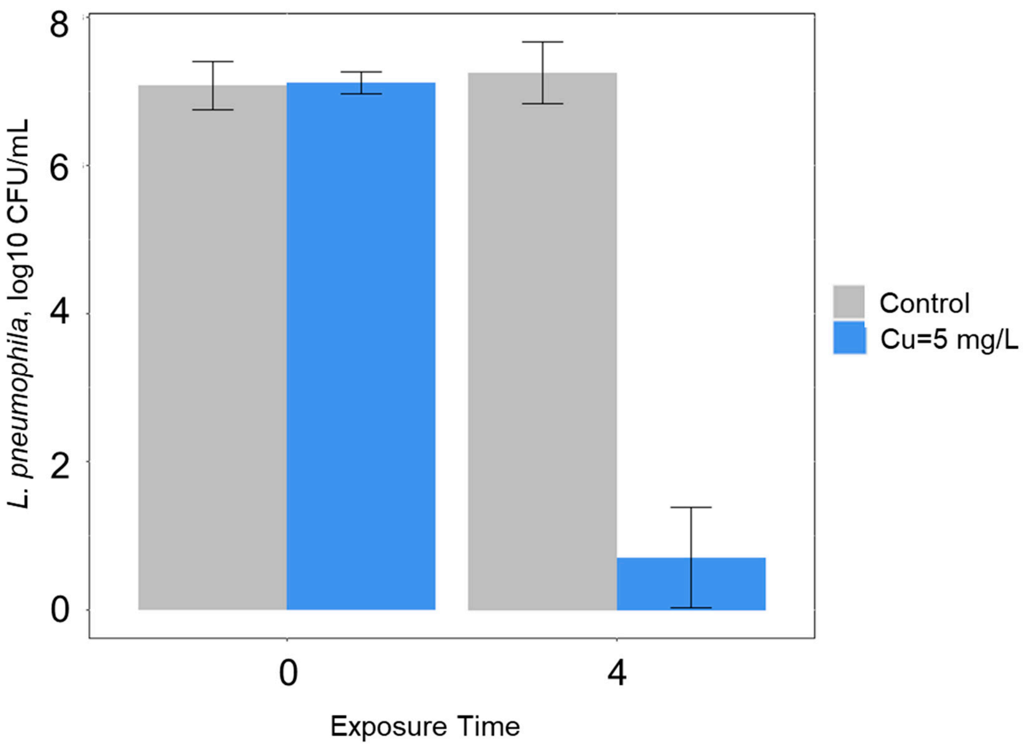

Figure S1. Copper inactivation experiments of *L. pneumophila* strain 130b culturability at time=0, and 4hrs after incubation at 37 °C with control (no copper dose) and Cu= 5 mg/L.

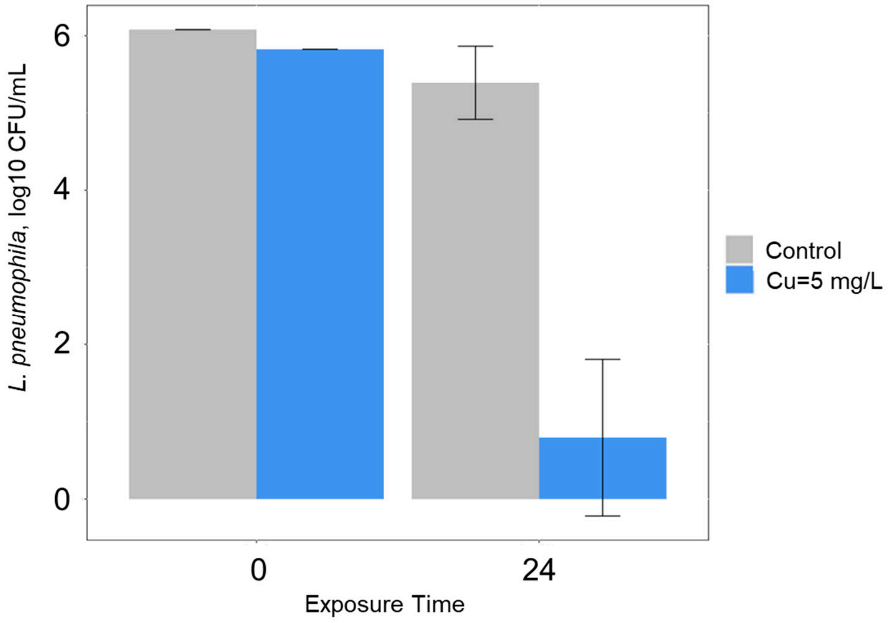

Figure S2. Copper inactivation experiments of *L. pneumophila* outbreak associated strain culturability at time=0, and 4hrs after incubation at 37 °C with control (no copper dose) and Cu= 5 mg/L.

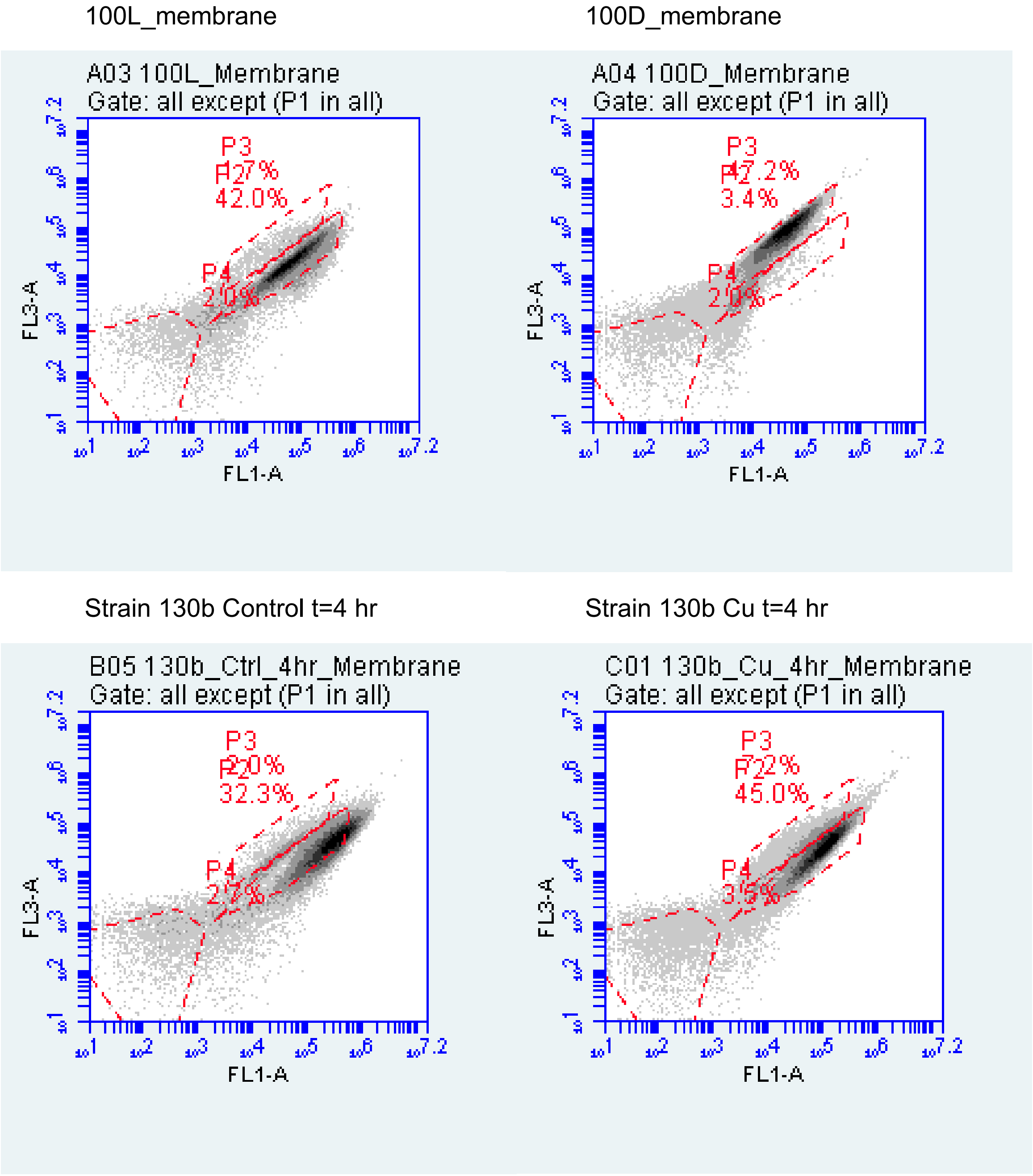

Figure S3. Example flowcytometry plots of *L. pneumophila* cell membrane integrity of A) 100% live strain 130b *L. pneumophila* cells; B) 100% dead strain 130b *L. pneumophila* cells after 70 °C heat treatment for one hour; C) control condition of strain 130b *L. pneumophila* cells after four hours incubation at 37 °C; D) Cu=5 mg/L condition of strain 130b *L. pneumophila* cells after four hours incubation at 37 °C. In the plot, gate P2 (right lower) represents viable cells and gate P3 (right upper) represents dead cells.

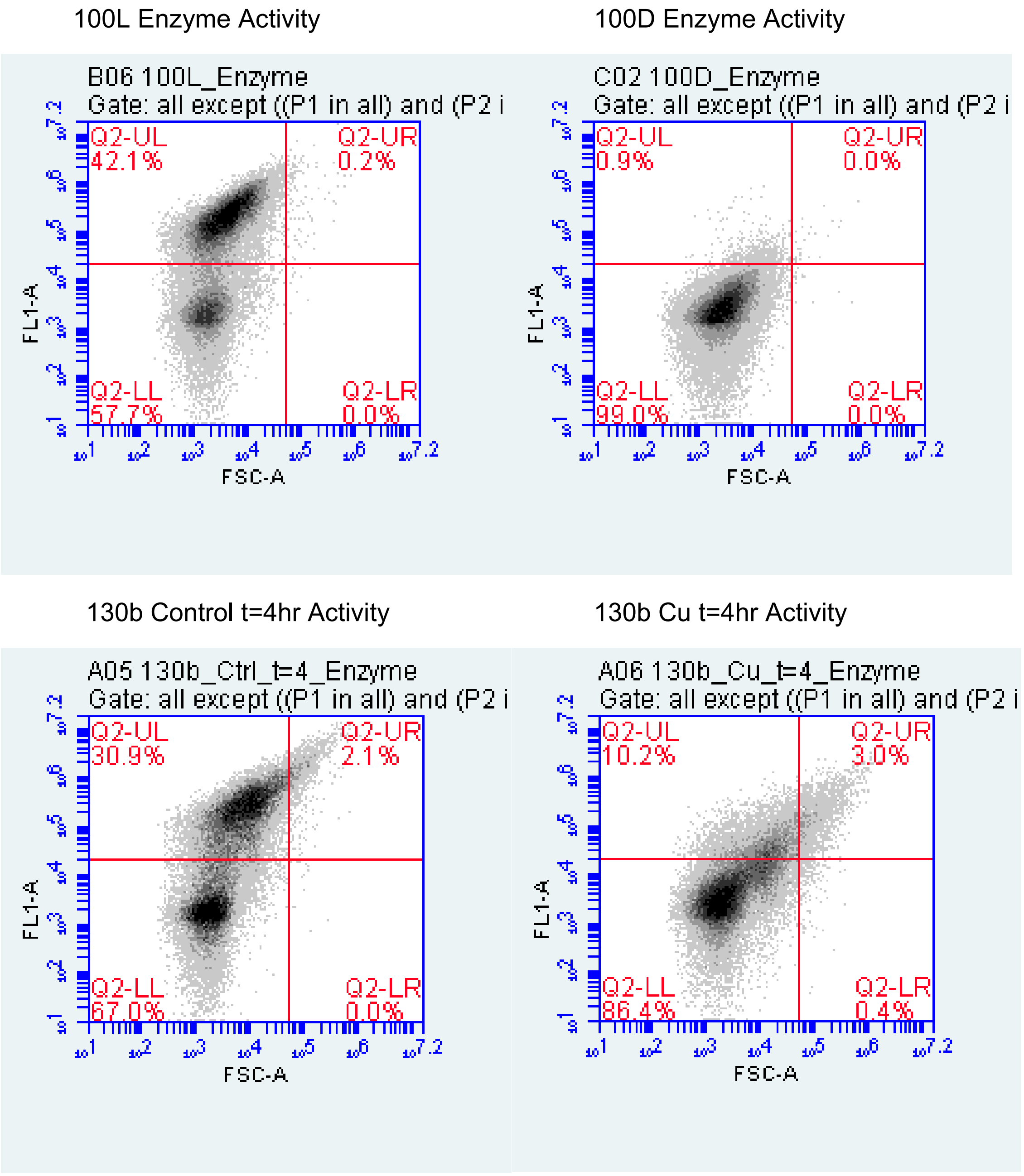

Figure S4. Example flowcytometry plots of *L. pneumophila* enzyme activity of A) 100% live strain 130b *L. pneumophila* cells; B) 100% dead strain 130b *L. pneumophila* cells after 70 °C heat treatment for one hour; C) control condition of strain 130b *L. pneumophila* cells after four hours incubation at 37 °C; D) Cu=5 mg/L condition of strain 130b *L. pneumophila* cells after four hours incubation at 37 °C. In the plot, quadrants UL and UR represent viable cells and quadrants LL and LR represent dead cells. As noted in the plot A, 100% live cells also rendered ~ 50% live signals, therefore a correction factor of 0.18 was applied to the dead cells signals across all the enzyme activities to counter the extra dead cells detection issue.

Table S1. ATP luminescence QAQC results

	Positive controls, μM ATP						Negative controls	
Condition	1E-10	1E-11	1E-12	1E-13	1E-14	1E-15	Dead cell blank	Background solution blank
Luminescence	3.1±1.6 E+5	3.6±2.1 E+4	3.2±2.2 E+3	3.1±2.5 E+2	1.0±1.4 E+2	1.9±1.5 E+1	2.0±2.3 E+2	1.5±0.6 E+1
# of samples	4	4	4	4	4	4	3	4

Table S2. Percent copper removal from cell pellets after treatments

Condition	Total Cu=1 mg/L; pH=7; *L. pneumophila* cell=10^6 CFU/mL	Total Cu=1 mg/L; pH=7; *L. pneumophila* cell=10^7 CFU/mL	Total Cu=2 mg/L; pH=7; *L. pneumophila* cell=10^6 CFU/mL	Total Cu=4 mg/L; pH=7; *L. pneumophila* cell=10^6 CFU/mL
Only spin-down	32.5%	61.3%	51.5%	58.4%
1X EDTA solution wash* and spin-down	87.0%	95.3%	87.9%	92.1%

* EDTA concentration was determined as 5:1 molar ratio of EDTA to copper with stock EDTA concentration of 0.5 M stored in 4 °C fridge.

Table S3. Identified proteins which are unique in copper exposed (a) and control (b) conditions at time=4hr.

(a) Proteins that are unique in copper exposed conditions.

Gene Family	Description	Gene.ontology.ID	Gene.ontology..GO.
A0A2S6F4I5 (obsolete, now Q5ZTL4, A0AA45B436)	Uncharacterized protein OS=Legionella pneumophila OX=446 GN=C3927_10720 PE=4 SV=1		
A0A130SXQ9 (obsolete, now A0AAD3U656)	Flagellar hook-associated protein 2 OS=Legionella pneumophila OX=446 GN=fliD PE=3 SV=1		
A0A129U261	Copper-translocating P-type ATPase OS=Legionella pneumophila OX=446 GN=C3927_10065 PE=3 SV=1	GO:0005524; GO:0005886; GO:0016021; GO:0019829; GO:0046872	integral component of membrane [GO:0016021]; plasma membrane [GO:0005886]; ATP binding [GO:0005524]; ATPase-coupled cation transmembrane transporter activity [GO:0019829]; metal ion binding [GO:0046872]
A0A2S6F6M3	SPOR domain-containing protein OS=Legionella pneumophila OX=446 GN=C3927_08635 PE=4 SV=1	GO:0042834	peptidoglycan binding [GO:0042834]
A0A4T1DZH2	Uncharacterized protein OS=Legionella pneumophila OX=446 GN=DIZ50_15865 PE=4 SV=1		
A0A2S6FC60 (obsolete, now Q5ZZ08, A0AA45B624)	DegV family EDD domain-containing protein OS=Legionella pneumophila OX=446 GN=C3927_00310 PE=4 SV=1	GO:0004371; GO:0006071	glycerone kinase activity [GO:0004371]; glycerol metabolic process [GO:0006071]
A0A4V4P644	ABC transporter permease OS=Legionella pneumophila OX=446 GN=DIZ50_07545 PE=3 SV=1	GO:0005886; GO:0016021; GO:0055085	integral component of membrane [GO:0016021]; plasma membrane [GO:0005886]; transmembrane transport [GO:0055085]
A0A129FBP0	Uncharacterized protein OS=Legionella pneumophila OX=446 GN=C3926_12120 PE=4 SV=1		
A0A378KHC2	Flagellar hook-associated protein 3 OS=Legionella pneumophila OX=446 GN=flgL PE=4 SV=1	GO:0005198; GO:0005576; GO:0009424; GO:0071973	bacterial-type flagellum hook [GO:0009424]; extracellular region [GO:0005576]; structural molecule activity [GO:0005198]; bacterial-type flagellum-dependent cell motility [GO:0071973]
A0A2S6F0Q3 (obsolete, now Q5ZRF0, A0AA45B3A6)	NAD(P)H-flavin reductase OS=Legionella pneumophila OX=446 GN=C3927_14810 PE=4 SV=1	GO:0016491	oxidoreductase activity [GO:0016491]

A0A130YDS6 (obsolete, now Q5ZTM5, Q5ZWQ8)	Cation efflux system protein CzcC OS=Legionella pneumophila OX=446 GN=czcC_1 PE=3 SV=1		
A0A378K256	Inner membrane protein yedI OS=Legionella pneumophila OX=446 GN=yedI PE=4 SV=1	GO:0016021	integral component of membrane [GO:0016021]
A0A2S6EVD4	RNA polymerase sigma factor RpoH OS=Legionella pneumophila OX=446 GN=rpoH PE=3 SV=1	GO:0001123; GO:0003677; GO:0005737; GO:0009408; GO:0016987	cytoplasm [GO:0005737]; DNA binding [GO:0003677]; sigma factor activity [GO:0016987]; response to heat [GO:0009408]; transcription initiation from bacterial-type RNA polymerase promoter [GO:0001123]
A0A4T1FZU7	ATP-binding protein OS=Legionella pneumophila OX=446 GN=DIZ50_03130 PE=4 SV=1	GO:0005524	ATP binding [GO:0005524]
A0A378K6C3	Chemiosmotic efflux system B protein A OS=Legionella pneumophila OX=446 GN=cusA_3 PE=3 SV=1	GO:0008324; GO:0016021	integral component of membrane [GO:0016021]; cation transmembrane transporter activity [GO:0008324]
A0A2S8C955 (obsolete, now G9EP55, A0A0W0UB68)	Porin OS=Legionella pneumophila OX=446 GN=C3926_10975 PE=3 SV=1	GO:0006811; GO:0008643; GO:0009279; GO:0015288; GO:0046930	cell outer membrane [GO:0009279]; pore complex [GO:0046930]; porin activity [GO:0015288]; carbohydrate transport [GO:0008643]; ion transport [GO:0006811]
A0A4V1ZI37	YceI family protein OS=Legionella pneumophila OX=446 GN=DIZ50_10045 PE=4 SV=1		
A0A2S8CCT9	Erythronate-4-phosphate dehydrogenase OS=Legionella pneumophila OX=446 GN=pdxB PE=3 SV=1	GO:0005737; GO:0008615; GO:0033711; GO:0051287	cytoplasm [GO:0005737]; 4-phosphoerythronate dehydrogenase activity [GO:0033711]; NAD binding [GO:0051287]; pyridoxine biosynthetic process [GO:0008615]
A0A4Q5NES5	Efflux pump membrane transporter OS=Legionella pneumophila OX=446 GN=DIZ50_04125 PE=3 SV=1	GO:0005886; GO:0015562; GO:0016021; GO:0042908	integral component of membrane [GO:0016021]; plasma membrane [GO:0005886]; efflux transmembrane transporter activity [GO:0015562]; xenobiotic transport [GO:0042908]
A0A130G112 (obsolete, now A0AAD3U1T9)	Uncharacterized protein conserved in bacteria OS=Legionella pneumophila OX=446 GN=ERS253249_01477 PE=4 SV=1		
A0A4T1FIM6	Uncharacterized protein OS=Legionella pneumophila OX=446 GN=DIZ50_12505 PE=4 SV=1		

A0A4T1FKZ3	CoA transferase OS=Legionella pneumophila OX=446 GN=DIZ50_11265 PE=4 SV=1	GO:0008410	CoA-transferase activity [GO:0008410]
A0A4T1EE93	AcrB/AcrD/AcrF family protein OS=Legionella pneumophila OX=446 GN=DIZ50_03895 PE=4 SV=1	GO:0016021; GO:0022857	integral component of membrane [GO:0016021]; transmembrane transporter activity [GO:0022857]
A0A4T1T0Y0 (obsolete, now Q5ZVG1)	Mechanosensitive ion channel protein MscS OS=Legionella pneumophila OX=446 GN=DI026_01080 PE=3 SV=1		
A0A130YUT4 (obsolete, now Q5ZUX8)	Penicillin-insensitive transglycosylase OS=Legionella pneumophila OX=446 GN=pbpD PE=3 SV=1		
A0A128YLG2 (obsolete, now A0AAD3YHM3)	Threonine synthase OS=Legionella pneumophila OX=446 GN=thrC PE=3 SV=1		
A0A4Q5NAG5	Protein-disulfide reductase OS=Legionella pneumophila OX=446 GN=DIZ50_06790 PE=3 SV=1	GO:0005886; GO:0016021; GO:0017004; GO:0047134	integral component of membrane [GO:0016021]; plasma membrane [GO:0005886]; protein-disulfide reductase (NAD(P)) activity [GO:0047134]; cytochrome complex assembly [GO:0017004]
A0A2S6F7I8 (obsolete, now Q5ZUX1, A0AA45B4X2)	Alpha-amylase OS=Legionella pneumophila OX=446 GN=C3927_07590 PE=4 SV=1	GO:0003824; GO:0005975	catalytic activity [GO:0003824]; carbohydrate metabolic process [GO:0005975]
A0A2S6F485	Type II/III secretion system protein OS=Legionella pneumophila OX=446 GN=C3928_03545 PE=4 SV=1	GO:0016020	membrane [GO:0016020]
A0A2S8C9K0	APH domain-containing protein OS=Legionella pneumophila OX=446 GN=C3926_10605 PE=4 SV=1		
A0A2S6FC95 (obsolete, now Q5ZZ01, A0AA44XM16)	Uncharacterized protein OS=Legionella pneumophila OX=446 GN=C3927_00345 PE=4 SV=1		
A0A4T1FY84	Uncharacterized protein OS=Legionella pneumophila OX=446 GN=DIZ50_06810 PE=4 SV=1	GO:0005085; GO:0007264	guanyl-nucleotide exchange factor activity [GO:0005085]; small GTPase mediated signal transduction [GO:0007264]

(b) Proteins that are unique in control conditions.

Gene Family	Description	Gene.ontology.ID	Gene.ontology..GO.
A0A4T1E5C9	Spermidine synthase OS=Legionella pneumophila OX=446 GN=DIZ50_12050 PE=4 SV=1		
A0A2S6F8T3 (obsolete, now Q5ZVK8, A0AA44XJY5)	FAD-binding oxidoreductase OS=Legionella pneumophila OX=446 GN=C3927_06390 PE=3 SV=1	GO:0016491; GO:0071949	FAD binding [GO:0071949]; oxidoreductase activity [GO:0016491]
A0A4T1E1M9	Type IV secretion protein Dot OS=Legionella pneumophila OX=446 GN=DIZ50_13370 PE=4 SV=1		
A0A378KBU2	Thymidine kinase OS=Legionella pneumophila OX=446 GN=tdk PE=3 SV=1	GO:0004797; GO:0005524; GO:0005737; GO:0008270; GO:0071897	cytoplasm [GO:0005737]; ATP binding [GO:0005524]; thymidine kinase activity [GO:0004797]; zinc ion binding [GO:0008270]; DNA biosynthetic process [GO:0071897]
A0A130QSM1 (obsolete, now A0AAD3YGY1)	DNA-binding transcriptional dual regulator Crp OS=Legionella pneumophila OX=446 GN=ERS253249_00899 PE=4 SV=1	GO:0003677; GO:0004222; GO:0016021; GO:0071586	integral component of membrane [GO:0016021]; DNA binding [GO:0003677]; metalloendopeptidase activity [GO:0004222]; CAAX-box protein processing [GO:0071586]
A0A4T1EHX5	Metal-binding protein OS=Legionella pneumophila OX=446 GN=DIZ50_02230 PE=4 SV=1		
A0A378KBD1	Small ribosomal subunit biogenesis GTPase RsgA OS=Legionella pneumophila OX=446 GN=rsgA PE=3 SV=1	GO:0003924; GO:0005525; GO:0005737; GO:0019843; GO:0042274; GO:0046872	cytoplasm [GO:0005737]; GTP binding [GO:0005525]; GTPase activity [GO:0003924]; metal ion binding [GO:0046872]; rRNA binding [GO:0019843]; ribosomal small subunit biogenesis [GO:0042274]
A0A4T1EHC3	Uncharacterized protein OS=Legionella pneumophila OX=446 GN=DIZ50_01240 PE=4 SV=1		

Table S4. Identified proteins with statistically significant increase of abundance in copper exposed (a) and control (b) conditions at time=4hr. Fold change was calculated dividing the test condition abundance over the comparison condition. LefSe analysis was used to run systematic comparison with $p<0.05$ and LDA>2.0 as threshold.

(a) Protein abundance increase associated with copper exposure

Accession	Fold change	LDA score	p-value
A0A2S8CE48 (obsolete, now Q5ZYR3)	2.988565824	2.13375492	0.049535
A0A131NJA7	3.929920572	3.2430033	0.049535
A0A2S6F779 (obsolete, now Q5ZV69, A0AA44XIL8)	3.139443876	2.46608473	0.049535
A0A378KP74	2.477648287	2.08370295	0.049535
A0A4T1FKE4	2.639299648	2.02982294	0.049535
A0A0C9P8T8	3.337129721	2.76190241	0.049535
A0A378KD96	2.572409062	2.14929413	0.049535
A0A129AHP7	2.732952842	2.13281705	0.049535
A0A378KHG7	3.231017641	2.67987745	0.049535
A0A4Q5NC14	3.109322206	2.27649801	0.049535
A0A4T1E313	3.146990755	2.56266643	0.049535
A0A131NB36	4.625383655	4.14769793	0.049535
A0A2S8CER9	2.703979068	2.05041659	0.049535
A0A130QZ98	3.069305521	2.54697496	0.049535
Q70YI1	4.089966981	3.30669852	0.049535
A0A4T1G1G2	3.112018378	2.57584802	0.049535
A0A128USX4	2.624777816	2.29303085	0.049535
A0A378KA29	2.649774637	2.03130551	0.049535
A0A2S6F4V3 (obsolete, now Q5ZTY2, A0AA44XH47)	3.486414648	2.75728758	0.049535
A0A133XCR1	3.444921101	2.25269143	0.049535
A0A4T1G3T5	2.654351497	2.10461657	0.049535
A0A4T1G1F3	2.659644881	2.2035958	0.049535
A0A130P7I5 (obsolete, now A0AAD3U165)	3.676128394	3.00633772	0.049535
A0A4T1FY84	0.090948619	2.16309872	0.036904
A0A4T1E5J5	3.072801843	2.4254691	0.049535
A0A128ZHV9	3.429832023	2.69824222	0.049535
A0A130QGT1	4.497817167	3.94780748	0.049535
A0A0C9MJM3	3.231456309	2.5138934	0.049535
A0A378KJA4	3.183429745	2.54984819	0.049535
A0A378K7D5	3.388712386	2.85386152	0.049535
A0A129G507	4.171895172	3.5136597	0.049535
A0A2S6FBT8	2.895980687	2.40066338	0.049535

A0A4Q5N7L5	2.721560541	2.28036955	0.049535
A0A0C9NYS4	3.171771588	2.22356145	0.049535
A0A128XJK1	2.721441883	2.19935546	0.049535
A0A128GUR2 (obsolete, now Q5ZV89)	3.02683008	2.42159852	0.049535
A0A4V1ZI00	3.42022913	2.74631329	0.049535
A0A378KJM0	3.120705044	2.66174803	0.049535
A0A4Q5N6Q2	3.550158563	2.78827088	0.049535
A0A378KG91	3.658892321	2.99944617	0.049535
A0A2S6ETC1 (obsolete, now A0A3A6UXS8)	3.363121555	2.817626	0.049535
A0A129DUW9	2.781756471	2.19317568	0.049535
A0A378KM53	2.705761939	2.18655668	0.049535
A0A2S6F921	3.384910907	2.80819762	0.049535
A0A0C9MU63	3.158546764	2.66578508	0.049535
A0A4T1E742	3.118724032	2.37575595	0.049535
A0A129XK80	3.162246954	2.22550513	0.049535
A0A0C9N7J0	3.28378052	2.70455449	0.049535
A0A128NM93	2.8231676	2.13285693	0.049535
A0A4T1FT42	3.190092585	2.44876535	0.049535
A0A0C9MAP6	2.980247713	2.31038253	0.049535
A0A378K3V4	3.758028938	2.96711914	0.049535
A0A2S6F5Y2	2.946196232	2.27364745	0.049535
A0A128ZWM7	3.904205218	3.17009227	0.049535
P26493	3.249783655	2.75369989	0.049535
A0A4T1EH95	2.686744839	2.02098938	0.049535
A0A130ZQ31	3.480586395	2.8131284	0.049535
A0A4Q5NEF4	2.971662344	2.35834915	0.049535
A0A378KFN1	2.682250608	2.00705887	0.049535
A0A4T1E469	2.83683915	2.34656213	0.049535
A0A4Q5N4N1	2.677956567	2.19537696	0.049535
A0A131MNJ4	3.645871577	2.83778339	0.049535
A0A4T1E4S5	3.879105972	3.20237584	0.049535
A0A128QX53	3.275377144	2.44079821	0.049535
A0A378KSI3	2.48698814	2.0341043	0.049535
A0A378KFM9	3.726181648	3.05465836	0.049535
A0A2S6EYY1	2.656940492	2.1133107	0.049535
A0A4T1FYK0	3.437096262	2.72936113	0.049535
A0A378KFL9	3.790938907	3.06201312	0.049535
W6CMK2	4.326056346	3.86572086	0.049535
A0A2S6FAT4 (obsolete, now A0AA44XL82)	3.094792608	2.47802897	0.049535
A0A0C9NJ73	3.398516275	2.60873889	0.049535
A0A0C9PWU0	3.140270606	2.46202028	0.049535

A0A0C9PUY8	3.22079929	2.58766407	0.049535
A0A0C9NUL1	3.101322473	2.5421082	0.049535
A0A128X5S9	3.406094675	2.71524847	0.049535
A0A128XDU8	3.615443392	3.1063512	0.049535
A0A4V4P653	3.281246146	2.67502417	0.049535
A0A130FYI2	3.35950284	2.43019137	0.049535
A0A128TSY4	2.602366866	2.12837887	0.049535
A0A4T1E6B3	3.106053411	2.45515948	0.049535
A0A131N4C8 (obsolete, now Q5ZXK0, Q5WYG8, A0AAD3YFP9, A0A3A6V4Z4)	2.762569667	2.12584676	0.049535
A0A129G4Y6 (obsolete, now Q5ZXJ9, A0A3A6WBM9)	3.306813983	2.71886459	0.049535
O85768	3.388885094	2.28688445	0.049535
A0A2S6EVA6	2.999380894	2.41472375	0.049535
A0A0C9PC59	3.522052477	2.930589	0.049535
A0A0C9PBN1	3.049943745	2.56462503	0.049535

(b) Protein abundance increase associated with control

Accession	Fold change	LDA score	p-value
A0A130WW92	4.13198607	3.39978753	0.049535
A0A4Q5N7T5	3.26046354	2.60494976	0.049535
A0A0C9MSH5	3.25995949	2.50447373	0.049535
A0A128LUL9	3.08862182	2.43273817	0.049535
A0A129SDS1	2.84588578	2.31233313	0.049535
A0A4T1EEJ8	2.79958033	2.18708456	0.049535
A0A4V1ZID9	3.0055342	2.45516306	0.049535
A0A130A4L3	3.93117581	3.22993498	0.049535
A0A2S6F2Z7 (obsolete, now Q5ZS94, A0AA44XIC6)	3.04404689	2.52423671	0.049535
A0A131NB48	3.14726049	2.56218047	0.049535
A0A4T1EHN6	3.01747326	2.41583931	0.049535
A0A378K866	2.86149171	2.26869437	0.049535
A0A128RQH4	2.97152935	2.25426904	0.049535
A0A4T1E925	2.9545242	2.18515555	0.049535
A0A0C9NJ87	4.37867639	3.63877874	0.049535
A0A4Q5N9G3	2.832892	2.22551221	0.049535
A0A4Q5NA35	3.33604394	2.81382564	0.049535
A0A4Q5NBE9	2.80555244	2.05871896	0.049535
A0A4Q5N5Q6	2.8248862	2.15850977	0.049535
A0A4V1ZIE4	3.08365272	2.42241691	0.049535
Q5C8M7	2.94985984	2.29494463	0.049535

A0A378KDT3	3.13830881	2.47882413	0.049535
A0A4Q5N9F5	2.62152831	2.02316618	0.049535
A0A2S8CER5	2.64874452	2.11718636	0.049535
A0A378KBK7	3.117016	2.37550534	0.049535
A0A4Q5NBB8	2.91419156	2.14789604	0.049535
A0A378KHA3	3.11431569	2.29124682	0.049535
A0A4Q5N7C4	2.70353605	2.08648173	0.049535
A0A0C9P9X4	3.57851578	2.63418386	0.049535
A0A4Q5N4W6	3.23382589	2.4172073	0.049535
A0A128WRR6 (obsolete, now A0AAD3YHJ5)	2.80907236	2.09569952	0.049535
A0A128QUV6	2.71325631	2.05926876	0.049535
A0A4T1E202	3.48138589	2.69811817	0.049535
A0A4T1E4Z4	3.09246984	2.37578709	0.049535
A0A130UX55	3.07860569	2.47897998	0.049535
A0A2S6F2U3	3.06843516	2.53629794	0.049535
A0A128YPA0	3.05915711	2.39566921	0.049535
A0A4Q5NAL2	3.04532232	2.38751506	0.049535
A0A378K4I7	3.66099293	3.23533186	0.049535
A0A130JZV5 (obsolete, now Q5ZWF9)	0	2.07161105	0.049535
A0A128R0D4	2.66285424	2.13912759	0.049535
A0A130RKW5	3.57007063	3.00022455	0.049535
A0A130QU18	3.01335884	2.48187115	0.049535
A0A4T1FPZ4	3.14173354	2.24001672	0.049535
A0A4V1ZI77	2.66712533	2.08741213	0.049535
A0A4V1ZIA5	2.8842623	2.17345357	0.049535
A0A0C9PF25	3.36234163	2.65138405	0.049535
A0A4V4P5Y3	3.22394654	2.72029586	0.049535
A0A378KFV9	3.37781586	2.71306619	0.049535
A0A4T1G0J1	3.1023391	2.25409433	0.049535
A0A4Q5NDR8	2.70852964	2.05545856	0.049535
A0A4T1SKR0 (obsolete, now Q5WZJ0, A5IHP4, Q5X837, Q5ZYM1)	3.56616365	2.8912957	0.049535
A0A128SQG8	3.55642362	2.93557417	0.049535
A0A128SR39	3.58653999	2.91754821	0.049535
A0A1K2H045 (obsolete, now Q5ZRV3)	3.48389013	2.84296452	0.049535
A0A4Q5N574	2.99337005	2.36923966	0.049535
A0A4T1ZLD0	3.03860818	2.44099324	0.049535
A0A4Q5N622	3.42246243	2.72200601	0.049535
A0A2U8UA06	2.72737667	2.15494972	0.049535
A0A128W7V2	2.70582752	2.01054185	0.049535

A0A4T1DX02	2.83272884	2.30402328	0.049535
A0A4Q5N961	3.76750405	2.93417478	0.049535
A0A131NPK7 (obsolete, now Q5ZXG3)	2.55694718	2.01028994	0.049535
A0A4T1ELJ0	2.69036353	2.08390371	0.049535
A0A4Q5NC69	2.62488792	2.20909602	0.049535
A0A0C9PND9	4.08401545	3.02619619	0.049535
A0A2S6ESY4	4.91421195	3.98559039	0.049535
A0A4T1F7Q0	2.94772895	2.32295242	0.049535
A0A378K5D3	2.92349045	2.08250835	0.049535
A0A129BID4	2.94936327	2.19023474	0.049535
A0A130GGP1	2.65093151	2.04070287	0.049535
A0A128UQH5	3.61318513	2.49549699	0.049535
A0A130CR05	2.61395664	2.06530682	0.049535
A0A378KJX4	3.34100037	2.69856646	0.049535
A0A130SVT8 (obsolete, now Q5ZRU1, Q5WT23)	3.35546398	2.48507403	0.049535
A0A0C9MH97	2.75123029	2.1134078	0.049535
A0A378KA59	2.99886699	2.29351111	0.049535
A0A378KD26	3.9093692	3.20339187	0.049535
A0A4T1FP43	3.73169386	3.01994881	0.049535
A0A378K9H6	4.10232207	3.30336444	0.049535
A0A378K3A8	2.89900734	2.31656511	0.049535
A0A131MJT8	2.80830037	2.19410884	0.049535
A0A4T1E0S3	3.00319917	2.43414917	0.049535
A0A378KDQ6	2.78641984	2.08260656	0.049535
A0A378KD55	3.95194184	3.29576118	0.049535
A0A378K8D0	3.02484404	2.49903142	0.049535
A0A4V4P5T4	3.03709158	2.42294446	0.049535
A0A4T1E715	2.87548081	2.14966796	0.049535
A0A378K0N5	3.3155383	2.44107074	0.049535
Q8RPF2	2.83960024	2.00175771	0.049535
A0A4Q5NDK6	3.23269671	2.50306099	0.049535
A0A130LVE0	3.06735675	2.3601319	0.049535
A0A128XGL5	2.67668087	2.02672488	0.049535
A0A4Q5N7X0	3.24087527	2.77287693	0.049535
A0A4V4P643	3.0981875	2.26350784	0.049535
A0A4Q5NAT3	3.31043508	2.43497055	0.049535
A0A4Q5NAJ7	2.67504538	2.05699574	0.049535
A0A128X4Q4	3.80765058	3.06777813	0.049535
Q8RPE9	2.80716022	2.05749839	0.049535
A0A128VVK7	3.37161903	2.46403387	0.049535
A0A128FUY8	3.14661647	2.46099363	0.049535

A0A129Z400	3.17776484	2.57472316	0.049535
A0A131LJT7	3.59213415	2.96402484	0.049535
Q8RNR8	3.85095619	2.67889035	0.049535
A0A4T1EHB0	2.94789073	2.25546763	0.049535
A0A4Q5NCM5	2.98207406	2.30367305	0.049535
P77890	3.23009697	2.45744428	0.049535
A0A4V1ZI91	3.12636216	2.55814691	0.049535
A0A131MZE3	2.80913189	2.15714618	0.049535
A0A130DD57 (obsolete, now Q5ZRU3)	3.40965107	2.39705145	0.049535

Table S5. Proteins related to copper, oxidoreductive process, and *Amoebae*/infection process detected in copper exposed (Cu_T4) and control (Control_T4) *L. pneumophila* strain 130b. Kruskal-Wallis tests were performed to compare protein abundance among triplicates of VBNC and culturable *L. pneumophila* and only the proteins showed statistically significant difference were indicated.

Category	Accession	Description	Cu_T4_1	Cu_T4_2	Cu_T4_3	Control_T4_1	Control_T4_2	Control_T4_3	p-value of Kruskal-Wallis tests
Cu related	A0A378KA59	Cytochrome c oxidase subunit 2 OS=Legionella pneumophila OX=446 GN=cyoA PE=3 SV=1	3.07E+08	2.47E+08	2.54E+08	5.16E+08	4.02E+08	3.09E+08	0.05
	A0A4T1E641	Multicopper oxidase family protein OS=Legionella pneumophila OX=446 GN=DIZ50_11635 PE=4 SV=1	3.67E+07	5.53E+07	8.62E+07	5.02E+07	5.94E+07	6.58E+07	
	A0A2S6ELT0	Cytochrome c oxidase assembly protein CtaG OS=Legionella pneumophila OX=446 GN=C3926_15785 PE=3 SV=1	1.19E+07	1.20E+07	2.99E+07	1.24E+07	1.38E+07	9.36E+06	
Oxidoreductive process	A0A4Q5N6Q2	Catalase-peroxidase OS=Legionella pneumophila OX=446 GN=katG PE=3 SV=1	1.13E+09	1.61E+09	1.63E+09	7.71E+08	8.53E+08	1.03E+09	
	A0A4Q5N7C4	Catalase-peroxidase OS=Legionella pneumophila OX=446 GN=katG PE=3 SV=1	1.23E+08	7.90E+07	1.16E+08	2.10E+08	2.35E+08	1.76E+08	
	A0A4T1Y461	Alkyl hydroperoxide reductase C OS=Legionella pneumophila OX=446 GN=DIZ50_05000 PE=3 SV=1	5.88E+07	4.55E+07	4.96E+07	9.89E+07	1.03E+08	9.29E+07	
	A0A4T1FF88	Organic hydroperoxide resistance protein OS=Legionella pneumophila OX=446 GN=DIZ50_15205 PE=3 SV=1	4.76E+07	5.09E+07	4.84E+07	3.28E+07	9.32E+07	4.72E+07	
	A0A4Q5NC85	Alkyl hydroperoxide reductase AhpD OS=Legionella pneumophila OX=446 GN=ahpD PE=3 SV=1	3.04E+07	3.54E+07	3.10E+07	1.55E+07	1.42E+07	1.51E+07	0.05
	A0A2S6F6D6	Thioredoxin reductase OS=Legionella pneumophila OX=446 GN=trxB PE=3 SV=1	3.64E+08	3.19E+08	3.28E+08	3.06E+08	2.62E+08	3.48E+08	
	A0A131CMU7 (obsolete, now Q5ZT15)	Alkyl hydroperoxide reductase C OS=Legionella pneumophila OX=446 GN=C3926_12210 PE=3 SV=1	NA	4.81E+06	4.25E+05	NA	3.60E+06	6.45E+06	
	A0A131NAA3	Superoxide dismutase OS=Legionella pneumophila OX=446 GN=sodB PE=3 SV=1	2.68E+08	3.10E+08	2.00E+08	2.17E+08	8.18E+07	2.62E+08	

	A0A378KKL8	Superoxide dismutase [Cu-Zn] OS=Legionella pneumophila OX=446 GN=DIZ50_04990 PE=3 SV=1	4.11E+07	4.13E+07	3.13E+07	2.06E+06	NA	3.35E+06	
Amoebae and infection process	Q9XD71	Type II secretion system core protein G OS=Legionella pneumophila OX=446 GN=lspG PE=3 SV=1	5.42E+07	7.27E+07	4.99E+07	4.05E+07	2.05E+07	4.42E+07	0.05
	A0A130TPH9	General secretion pathway protein F OS=Legionella pneumophila OX=446 GN=epsF_2 PE=3 SV=1	1.19E+07	1.09E+07	8.30E+06	1.35E+07	9.97E+06	7.89E+06	
	A0A4T1FVD6	Type II secretion system protein L OS=Legionella pneumophila OX=446 GN=DIZ50_07745 PE=3 SV=1	9.41E+06	1.81E+06	1.48E+06	1.55E+07	1.54E+07	2.59E+05	
	A0A2S6F6M2 (obsolete, now Q5ZUC9, A0AA45B506)	Type II secretion system protein M OS=Legionella pneumophila OX=446 GN=C3927_08630 PE=3 SV=1	9.62E+06	1.38E+07	1.51E+07	1.39E+07	8.09E+06	1.10E+07	
	A0A378K550	Type IV pilus assembly protein PilA OS=Legionella pneumophila OX=446 GN=pilA PE=3 SV=1	8.19E+08	6.70E+08	8.79E+08	9.02E+08	7.11E+08	7.70E+08	
	A0A2S6EYN5	Type II secretion system protein L OS=Legionella pneumophila OX=446 GN=C3928_09430 PE=3 SV=1	2.59E+04	2.57E+04	1.71E+04	NA	3.01E+04	7.36E+04	0.05
	A0A130SA60 (obsolete, now A0AAD3U5C1)	Type II secretion system protein J OS=Legionella pneumophila OX=446 GN=xcpW PE=3 SV=1	1.38E+05	8.59E+04	NA	1.06E+05	NA	NA	
	A0A2S6EYB2	H(+)-transporting two-sector ATPase OS=Legionella pneumophila OX=446 GN=fliI PE=3 SV=1	NA	1.79E+06	1.54E+06	3.46E+06	3.46E+06	NA	
	Q8RPE1	Probable conjugal transfer protein OS=Legionella pneumophila OX=446 GN=lvhB6 PE=4 SV=1	1.81E+07	6.57E+06	9.50E+06	4.58E+07	1.80E+07	2.28E+06	
	Q8RPD7	Type IV secretion system protein OS=Legionella pneumophila OX=446 GN=lvhB11 PE=3 SV=1	1.24E+07	1.12E+07	1.57E+07	2.12E+07	1.02E+07	5.79E+06	
	A0A4V4QXT8	Type IV secretion system protein OS=Legionella pneumophila OX=446 GN=virB11 PE=3 SV=1	5.16E+07	4.76E+07	4.67E+07	5.87E+07	6.29E+07	4.16E+07	
